# Associations of Monocytes and the Monocyte/High-Density Lipoprotein Ratio With Extracranial and Intracranial Atherosclerotic Stenosis

**DOI:** 10.3389/fneur.2021.756496

**Published:** 2021-12-03

**Authors:** Zhu Liu, Qingli Fan, Shizheng Wu, Yancheng Lei

**Affiliations:** Qinghai Provincial People's Hospital, Xining, China

**Keywords:** inflammation markers, atherosclerotic stenosis, DSA (digital subtraction angiogram), lipoprotein, cerebral arteries

## Abstract

**Background:** Although the monocyte/high-density lipoprotein ratio (MHR) has been shown to be a potential marker of inflammatory of cardiovascular and cerebrovascular diseases, there are few studies on its relationships with the degree of intracranial and extracranial atherosclerotic stenosis and the stenosis distribution.

**Methods:** In total, 271 patients were admitted for digital subtraction angiography (DSA) examination and were classified into a non-stenosis group and a stenosis group. (1) The two groups were compared and the arteries were categorized according to the degree of intracranial or extracranial atherosclerotic stenosis (if ≥two branches were stenotic, the artery with the most severe stenosis was used). (2) Clinical baseline data and laboratory indexes of patients grouped according to stenosis location (intracranial vs. extracranial) were collected.

**Results:** (1) MHR × 10^2^ [odds ratio (OR) = 1.119, *p* < 0.001], age (OR = 1.057, *p* = 0.007), and lymphocyte count (OR = 0.273, *p* = 0.002) significantly affected the presence of cerebral atherosclerotic stenosis, with an MHR area of 0.82 under the receiver operating characteristic (ROC) curve (AUC) and an optimal diagnostic value of 0.486. Analyses of the moderate, mild, and severe stenosis groups showed that MHR × 10^2^ (OR = 1.07, *p* < 0.001) significantly affected the severity of stenosis in patients. (2) In the analysis of stenosis at different sites, the rate of extracranial artery stenosis in patients who smoked (OR = 3.86, *p* = 0.023) and had a reduced lymphocyte level (OR = 0.202, *p* = 0.001) was remarkably greater than that in patients who smoked (OR = 3.86, *p* = 0.023). With increasing age, the rate of extracranial artery stenosis raised sharply. With the increase in the MHR level, the stenosis rate of each group was highly greater than that of the non-stenosis group.

**Conclusion:** The MHR has a predictive value for the diagnosis of extracranial and intracranial atherosclerotic stenosis and is correlated with the degree and distribution of stenosis.

**Trial Registration:** Clinical Medical Research Center Project of Qinghai Province (2017-SF-L1). Qinghai Provincial Health Commission Project (Grant #2020-wjzdx-29).

## Background

Atherosclerosis is a common chronic illness characterized by endovascular atheroma or fibrous plaque. Pathophysiological changes, namely, arterial wall hardening, decreased elasticity, and lumen stenosis or occlusion, are important risk factors for the occurrence and development of ischemic cerebrovascular diseases and mortality (1, 2). Inflammatory factors play an essential role in lipid metabolic disorders and their importance in thrombosis, plaque rupture, and stenosis in atherosclerosis is being increasingly reported (3). Studies on the correlations of the monocyte/high-density lipoprotein ratio (MHR) [determined *via* dividing the absolute monocyte count by the absolute high-density lipoprotein cholesterol (HDL-C) count] with coronary stenosis and myocardial infarction have shown that the MHR is a potential inflammatory marker of cerebrovascular and cardiovascular diseases (4, 5). Currently, there are few reports on cerebrovascular diseases and most are relevant to the occurrence and prognosis of ischemic stroke (6, 7). Digital subtraction angiography (DSA) is regarded as a perfect standard for diagnosing intracranial and extracranial arterial stenosis. However, studies using DSA to investigate the relationship between intracranial or extracranial arterial stenosis and the MHR are rarely reported. This study investigated the relationships between the MHR and intracranial and extracranial arterial stenosis and related risk factors, aiming to proposed a reliable theoretical foundation to guide the treatment and prevention of intracranial and extracranial arterial stenosis.

## Methods

### Study Population

From May 2017 to May 2020, a total of 216 inpatients with intracranial and extracranial atherosclerotic stenosis confirmed by cerebrovascular DSA examination at the Qinghai Provincial People's Hospital were consecutively enrolled. There were 55 hospitalized patients without intracranial and extracranial atherosclerotic stenosis. DSA examination included the aortic arch, subclavian artery, vertebral artery, common carotid artery, and internal carotid artery. The common carotid artery, intracranial internal carotid artery (cervical segment, petrous segment, lacerum segment, and so on), V1–V3 segments of the vertebral artery, subclavian artery, and external carotid artery are classified as extracranial arteries. Intracranial arteries include the extracranial internal carotid artery (ophthalmic segment and communicating segment), A1–A2 segments of the anterior cerebral artery, basilar artery, M1–M2 segments of the middle cerebral artery, P1–P2 segments of the posterior cerebral artery, and V4 segment of the vertebral artery. The exclusion criteria were as follows: acute cardiovascular disease, signs of acute infection, immunosuppressive therapy, tumor, hematological system disorder, connective tissue disease, severe liver and kidney function impairment, moyamoya disease, and arteriovenous malformation. The Qinghai Provincial People's Hospital Ethics Committee approved this study.

### Blood Analysis Methods

Basic clinical data, such as gender, age, ethnicity, hypertension, drinking, smoking, diabetes mellitus, and cerebral infarction, were collected from patients meeting the inclusion criteria. Additionally, laboratory measures of monocytes, HDL-C, and the MHR of patients were obtained within 24 h after admission (with monocytes and HDL-C measured from the same initial blood sample). (1) First, the stenosis and non-stenosis groups were compared. Then, the DSA examination results were assessed based on relevant diagnostic criteria developed for the Warfarin-Aspirin Symptomatic Intracranial Disease Study (8) to evaluate the content of intracranial and extracranial atherosclerotic stenosis, which was calculated as follows: Degree of stenosis (%) = (1-diameter at the narrowest point of a narrow segment/the diameter of the proximal normal vessel) × 100%. According to the degree of arterial stenosis, the patients were divided into a mild stenosis group (stenosis degree of 29% or less), a moderate stenosis group (stenosis degree of 30–69%), and a severe stenosis group (stenosis degree of 70–99%). The aim was to study the factors influencing the degree of atherosclerotic stenosis and to analyze the predictive value of the MHR for cerebral atherosclerotic stenosis. (2) Based on the location of extracranial and intracranial atherosclerotic stenosis, the patients were divided into four groups: a non-stenosis group, an intracranial atherosclerosis only group (ICAS group), an extracranial atherosclerosis only group (ECAS group), and an intracranial and extracranial atherosclerosis group (I-ECAS group). The non-stenosis group worked as a control group and was compared with the other three groups to analyze influencing factors.

### Statistical Analyses

The SPSS software version 26.0 (Chicago, Illinois, USA) was employed for the analysis of the data. The chi-squared test was used for count data. The experimental data were examined for normality by the Shapiro–Wilk normality test; those with normal distributions were expressed as the mean ± SD and analyzed with the one-way ANOVA. The characteristics of baseline of the non-stenosis group and stenosis group were compared with the Mann–Whitney *U*-test. For data distributed non-normally, the data were expressed as medians (lower quartile-upper quartile) and analyzed with non-parametric tests. The predictive power of the MHR for the occurrence of cerebral atherosclerotic stenosis was analyzed using the subject working characteristic curve and the optimal threshold was determined. Finally, variables with statistical significance (*p* < 0.05) in the univariate analyses were involved within the logistic regression model.

## Results

### Analysis of Logistic Regression and Receiver Operating Characteristic Curve for Cerebral Artery Stenosis

**Figure 1:**
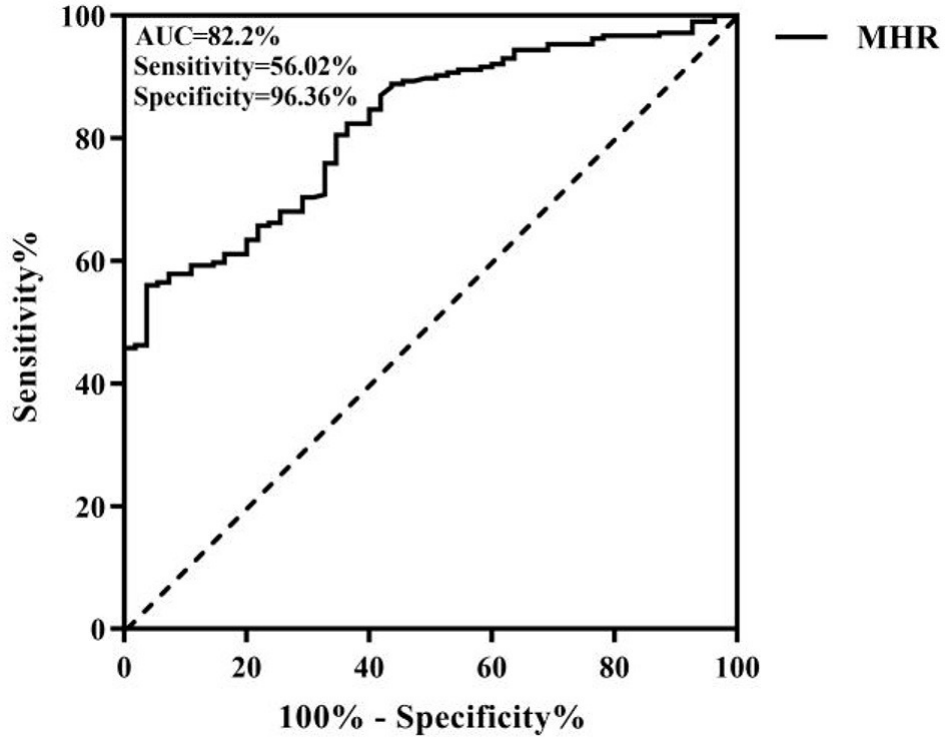
Receiver operating characteristic (ROC) curve of the monocyte/high-density lipoprotein ratio (MHR) for intracranial and extracranial atherosclerotic stenosis.

Age, neutrophil count, white blood cell (WBC) count, the MHR, C-reactive protein (CRP), proportion of males, smoking, drinking, hypertension, and diabetes mellitus were greater within the stenosis group (*n* = 216) than in the non-stenosis group (*n* = 55) and lymphocyte count was lower in the non-stenosis group; these differences were remarkable (*p* < 0.05). In the univariate and multivariable logistic regression analyses, age was the independent variable found to be positively associated with the probability of narrow stenosis [*p* = 0.007 < 0.05, odds ratio (OR) = 1.057 > 1]. A higher MHR × 10^2^ value was associated with a greater probability of stenosis (*p* < 0.001, OR = 1.119 > 1) and a higher lymphocyte count was associated with a lower probability of stenosis (*p* = 0.002 < 0.05, OR = 0.273 < 1) as shown in Table 1. The ROC curve analysis of the MHR and cerebral atherosclerotic stenosis yielded an area under the ROC curve (AUC) of 0.82 and the optimal diagnostic value was 0.486; the results are plotted in Figure 1.

**Table 1:** Comparison of factors between the non-stenosis group and the atherosclerotic stenosis group.

**Variable**	**Univariate analysis**		**Z/c^2^**	***P***	**Multifactor logistic regression**		
	**Non-stenosis group (*n* = 55)**	**Stenosis group (*n* = 216)**			**OR**	**95% CI**	***P***
Age	53 (42–61)	62 (52–69)	−4.715	<0.001	1.057	1.016–1.1	0.007
Sex, male	19 (11.2%)	150 (88.8%)	22.747	<0.001	0.733	0.286–1.879	0.518
Smoking	12 (12.1%)	87 (87.9%)	6.443	0.011	1.129	0.425–2.998	0.807
Drinking	2 (4.3%)	45 (95.7%)	9.044	0.003	2.353	0.4–13.835	0.344
Hypertension	25 (14.3%)	150 (85.7%)	11.029	0.001	0.994	0.397–2.487	0.989
Diabetes mellitus	4 (6%)	63 (94%)	11.291	0.001	2.702	0.734–9.945	0.135
Acute stroke/TIA	10 (18.2%)	136 (63.0%)	40.305	<0.001	6.800	3.323–13.916	<0.001
WBC count	5.21 (4.43–6.62)	6.4 (5.47–7.63)	−3.949	<0.001	1	0.921–1.086	0.997
Neutrophil count	3.22 (2.24–3.83)	4.16 (3.24–5.28)	−4.916	<0.001	1.313	0.966–1.785	0.083
Lymphocyte count	1.83 (1.39–2.23)	1.62 (1.23–1.96)	−2.563	0.01	0.273	0.119–0.624	0.002
PLT	195 (160–253)	186.5 (144–227.5)	−1.92	0.055^a^	0.998	0.99–1.005	0.551
CRP	1.27 (0.56–2.45)	2.28 (1.07–5.63)	−3.872	<0.001^b^			
TC	4.27 (3.43–5.14)	4.21 (3.64–4.86)	−0.053	0.958			
TG	1.38 (1.08–1.92)	1.55 (1.09–2.24)	−1.095	0.274			
LDL	2.54 (1.95–3.3)	2.49 (2–3.08)	−0.233	0.816			
Apolipoprotein A	1.19 (1.1–1.47)	1.16 (1.05–1.31)	−1.741	0.082^a^	2.302	0.362 14.636	0.377
Apolipoprotein B	0.85 (0.66–1.05)	0.91 (0.75–1.04)	−1.215	0.224			
MHR × 10^2^	0.31 (0.25–0.43)	0.5 (0.39–0.58)	−7.366	<0.001	1.119	1.07–1.17	**<0.001**

a *p < 0.05 as assessed by the univariate logistic regression; variable was subsequently included in the multivariate analysis.*

b *p > 0.05 as assessed by the univariate logistic regression; variable was not included in the multivariate analysis.*

*WBC, white blood cell; PLT, blood platelets; CRP, C-reactive protein; TC, total cholesterol; TG, triglyceride; LDL, low-density lipoprotein.*

*Bold value indicates statistical analysis methods and groupings*.

Patients with mild (*n* = 72), moderate (*n* = 35), and severe (*n* = 60) stenosis were selected for analysis and the group differences in WBC count, neutrophil count, CRP, apolipoprotein A, and the MHR were significant (*p* < 0.05). The variables found to be significant in the univariate ordered logistic regression analysis were included in the multivariate ordered logistic regression analysis, with the severity of stenosis as the dependent variable. The results showed that the MHR alone significantly influenced the severity of stenosis (*p* < 0.001, OR = 1.07 > 1), i.e., the greater the MHR value was, the greater the stenosis severity was, as shown in Table 2.

**Table 2:** Associations of factors with the severity of intracranial atherosclerotic stenosis.

**Variable**	**Univariate analysis**			**F/c^2^/Z**	***P***	**Multifactor logistic regression**		
	**Mild stenosis (*n* = 72)**	**Moderate stenosis (*n* = 35)**	**Severe stenosis (*n* = 60)**			**OR**	**95% CI**	***P***
Age	62.76 ± 12.48	60.49 ± 13.14	63.37 ± 10.67	0.666	0.515			
Sex, Male	46 (39.7%)	23 (19.8%)	47 (40.5%)	−1.747	0.081			
Smoking	22 (34.4%)	16 (25%)	26 (40.6%)	−1.565	0.118			
Drinking	12 (36.4%)	8 (24.2%)	13 (39.4%)	−1.565	0.118			
Hypertension	54 (45.4%)	24 (20.2%)	41 (34.5%)	−0.865	0.387			
Diabetes mellitus	16 (30.8%)	11 (21.2%)	25 (48.1%)	−2.392	0.017	1.74	0.882–3.435	0.11
Acute stroke/TIA	35 (48.6%)	12 (34.3%)	47 (78.3%)	12.274	0.002	2.57	1.428–4.609	0.002
WBC count	5.92 ± 1.47	6.74 ± 1.39	6.77 ± 1.58	6.404	<0.001	1.063	0.588–1.919	0.84
Neutrophil count	3.56 (2.9–4.59)	4.23 (3.44–5.23)	4.15 (3.45–5.46)	8.431	0.015	1.052	0.591–1.876	0.862
Lymphocyte count	1.51 ± 0.51	1.59 ± 0.55	1.69 ± 0.51	2.005	0.138			
PLT	171.17 ± 52.47	182.83 ± 43.45	186.48 ± 58.51	1.472	0.232			
CRP	1.21 (0.74–2.29)	2.24 (1.23–4.42)	3.27 (2.1–8.07)	30.119	<0.001^a^			
TC	4.2 (3.66–4.85)	4.4 (3.56–5.2)	4.18 (3.7–4.79)	0.611	0.737			
TG	1.38 (0.97–1.84)	1.55 (1.16–2.32)	1.9 (1.18–2.57)	5.555	0.062			
LDL	2.48 (2.03–3.02)	2.69 (1.79–3.5)	2.55 (2.06–3.03)	0.665	0.717			
Apolipoprotein A	1.21 (1.11–1.39)	1.24 (1.1–1.39)	1.16 (1.01–1.25)	7.504	0.023^a^			
Apolipoprotein B	0.91 (0.77–0.99)	0.89 (0.7–1.1)	0.92 (0.78–1.05)	0.493	0.782			
MHR × 10^2^	0.4 (0.28–0.51)	0.47 (0.38–0.55)	0.55 (0.45–0.68)	39.392	<0.001	1.07	1.043–1.1	**<0.001**

a *p > 0.05 as assessed by the univariate logistic regression; variable was not included in the multivariate analysis. p = 0.784 > 0.05 for the ordered logistic parallel line test.*

*Bold value indicates statistical analysis methods and groupings*.

### Analysis of the Factors Correlated With the Distribution of Atherosclerotic Stenosis in Intracranial and Extracranial Arteries

There were remarkable differences in sex, smoking, drinking, diabetes mellitus, hypertension, WBC count, neutrophil count, lymphocyte count, CRP, low-density lipoprotein (LDL), apolipoprotein A, and the MHR among the atherosclerosis-free, ICAS, ECAS, and I-ECAS groups (*p* < 0.05), as shown in Table 3.

**Table 3:** Associations of factors with the distribution of intracranial atherosclerotic stenosis.

**Variable**	**No stenosis (*n* = 55)**	**Intracranial atherosclerosis alone (*n* = 64)**	**Extracranial atherosclerosis alone (*n* = 115)**	**Combined intracranial and extracranial atherosclerosis (*n* = 37)**	**F/c^**2**^**	***P***
Age	50.91 ± 12.56	56.17 ± 11.6	61.59 ± 13.53	65.16 ± 8.5	13.752	<0.001
Sex, male	19 (11.2%)	39 (23.1%)	83 (49.1%)	28 (16.6%)	25.697	<0.001
Smoking	8 (8.1%)	29 (19.2%)	59 (59.6%)	13 (13.1%)	23.615	<0.001
Drinking	2 (4.3%)	14 (29.8%)	22 (46.8%)	9 (19.1%)	9.639	0.022
Hypertension	25 (14.3%)	43 (24.6%)	77 (44%)	30 (17.1%)	13.673	0.003
Diabetes mellitus	4 (6%)	19 (28.4%)	30 (44.8%)	14 (20.9%)	13.381	0.004
Acute stroke/TIA	10 (18.2%)	44 (68.8%)	73 (63.5%)	19 (51.4%)	38.259	<0.001
WBC count	5.21 (4.43–6.62)	6.73 (5.39–8.31)	6.25 (5.47–7.37)	6.54 (5.41–7.75)	17.34	0.001
Neutrophil count	3.22 (2.24–3.83)	4.24 (3.17–5.38)	4.13 (3.23–5.03)	4.09 (3.29–5.6)	24.742	<0.001
Lymphocyte count	1.83 (1.39–2.23)	1.71 (1.28–2.11)	1.57 (1.19–1.88)	1.65 (1.29–2.19)	11.11	0.011
PLT	195 (160–253)	194.5 (148–252)	172 (139–218)	191 (143.5–210.5)	7.503	0.057^a^
CRP	1.27 (0.56–2.45)	2.33 (1.27–6.54)	2.2 (0.99–5.63)	2.25 (1.23–5.24)	15.177	0.002^b^
TC	4.27 (3.43–5.14)	4.33 (3.66–4.92)	4.04 (3.53–4.66)	4.62 (3.88–5.22)	5.166	0.16
TG	1.38 (1.08–1.92)	1.55 (1.06–2.15)	1.51 (1.16–2.25)	1.6 (1.11–2.31)	1.818	0.611
LDL	2.54 (1.95–3.3)	2.56 (2.12–3.3)	2.37 (1.9–2.83)	2.81 (2.31–3.43)	8.604	0.035^b^
Apolipoprotein A	1.19 (1.1–1.47)	1.22 (1.11–1.43)	1.16 (1.04–1.28)	1.1 (1–1.3)	12.021	0.007
Apolipoprotein B	0.85 (0.66–1.05)	0.92 (0.75–1.06)	0.88 (0.74–1.01)	0.93 (0.8–1.11)	3.898	0.273
MHR × 10^2^	0.31 (0.25–0.43)	0.46 (0.38–0.55)^c^	0.5 (0.38–0.58)^c^	0.55 (0.43–0.7)^c^	59.049	<0.001

a *p < 0.05 as assessed by the univariate logistic regression; variable was subsequently included in the multivariate analysis.*

b *p > 0.05 as assessed by the univariate logistic regression; variable was not included in the multivariate analysis.*

c *There was a significant difference between the two groups (p < 0.05)*.

### Logistic Regression Analysis of Atherosclerotic Stenosis Distribution in Intracranial and Extracranial Arteries

Following the univariate ordered logistic regression analysis, the multivariate ordered logistic regression analysis was conducted, which excluded CRP and LDL, but included platelets. The results suggested that age was significantly and positively associated with the probability of simple extracranial stenosis (*p* = 0.003 < 0.05, OR = 1.066 > 1) and the probability of combined intracranial and extracranial atherosclerotic stenosis (*p* < 0.001, OR = 1.102 > 1). Smoking (*p* = 0.023 < 0.05, OR = 3.86 > 1) significantly increased the incidence of simple extracranial atherosclerotic stenosis. The higher the lymphocyte value was, the lower was the probability of developing simple extracranial atherosclerotic stenosis (*p* = 0.001 < 0.05, OR = 0.202 < 1). A higher MHR × 10^2^ value was associated with higher probabilities of simple intracranial atherosclerotic stenosis (*p* < 0.001, OR = 1.12 > 1), simple extracranial atherosclerotic stenosis (*p* < 0.001, OR = 1.121 > 1), and combined intracranial and extracranial atherosclerotic stenosis (*p* < 0.001, OR = 1.147 > 1), as shown in Table 4.

**Table 4:** The multivariate logistic regression analysis of the distribution of intracranial atherosclerotic stenosis.

**Variable**	**ICAS**		**ECAS**		**I-ECAS**	
	**OR (95% CI)**	***P***	**OR (95% CI)**	***P***	**OR (95% CI)**	***P***
Age	1.032 (0.987–1.078)	0.166	1.066 (1.022–1.113)	0.003	1.102 (1.045–1.162)	<0.001
Sex, Male	1.003 (0.336–2.994)	0.996	1.149 (0.409–3.229)	0.792	1.781 (0.469–6.759)	0.396
Smoking	1.347 (0.381–4.76)	0.644	3.86 (1.206–12.357)	0.023	1.119 (0.274–4.571)	0.875
Drinking	2.793 (0.417–18.706)	0.29	1.094 (0.172–6.964)	0.924	2.222 (0.292–16.937)	0.441
Hypertension	1.054 (0.376–2.958)	0.92	0.748 (0.28–1.996)	0.562	1.467 (0.408–5.269)	0.557
Diabetes mellitus	3.507 (0.877–14.023)	0.076	2.202 (0.563–8.608)	0.256	3.114 (0.7–13.856)	0.136
Acute stroke/TIA	9.900 (4.167–23.522)	<0.001	7.821 (3.573–17.119)	<0.001	4.750 (1.854–12.170)	0.001
WBC count	0.697 (0.094–5.195)	0.725	1 (0.906–1.103)	0.998	0.309 (0.018–5.317)	0.418
Neutrophil count	1.941 (0.245–15.35)	0.53	1.237 (0.89–1.719)	0.206	4.197 (0.228–77.345)	0.335
Lymphocyte count	0.467 (0.043–5.074)	0.531	0.202 (0.081–0.501)	0.001	1.243 (0.045–34.472)	0.898
PLT	0.999 (0.99–1.007)	0.8	0.997 (0.989–1.005)	0.483	0.998 (0.988–1.008)	0.664
MHR × 10^2^	1.12 (1.067–1.176)	<0.001	1.121 (1.07–1.174)	<0.001	1.147 (1.087–1.21)	<0.001
Apolipoprotein A	4.903 (0.688–34.939)	0.113	1.085 (0.139–8.488)	0.938	2.202 (0.155–31.384)	0.56

## Discussion

Atherosclerosis is a common chronic inflammatory disease. Inflammation is an important pathophysiological mechanism of atherosclerotic thrombosis, plaque rupture, and stenosis or occlusion. Monocytes are immune cells and when the vascular endothelium is damaged, the expression of adhesion molecules upon the surface of these cells increases. Upon stimulation by cytokines, these cells transform into macrophages. Phagocytosis of lipids occurs followed by the formation of foam cells under scavenger receptor mediation; these changes mark the initial phase of atherosclerosis and the transition from a stable to an unstable state. The lipid core of atherosclerotic lesions contains not only lipid deposits, but also a variety of immune cells derived from monocytes and macrophages that include T cells, mast cells, and dendritic cells, which act as major roles within the proliferation and progression of atherosclerosis (9, 10). Monocytes can aggravate inflammation and promote the development and instability of plaques, local thrombosis, and a series of responses, thus aggravating vascular stenosis. Dyslipidemia is another significant risk factor for atherosclerosis. The main function of HDL-C is the reverse transport of total cholesterol in the tissues toward the liver and out of the body. HDL-C can reduce thrombosis risk *via* platelet stabilization and decrease leukocyte adhesion to stable plaques. HDL-C can also prevent LDL oxidation and exhibit antithrombotic and anti-inflammatory properties, thereby playing a protective role (11, 12). Study has revealed great prospects of HDL-C infusion for the treatment of atherosclerosis (13, 14). Monocytes are closely related to HDL-C. Abnormal levels of blood lipids, especially elevated cholesterol, can stimulate the production of monocytes in the circulation. Furthermore, reduced HDL-C can reduce the monocyte inflammatory response (15). The ability of monocytes to phagocytose lipid particles is enhanced in atherosclerotic stenosis, making blood fat more likely to be deposited in the stenosis (16). Therefore, it is speculated that the MHR has more advantages than monocytes and HDL-C as an inflammatory marker. This study found that the MHR is an independent factor of risk for the occurrence of cerebral atherosclerosis. ROC curve analysis showed that the area under the ROC curve (AUC) of the MHR was 0.82 and the optimal diagnostic value was 0.486, showing that the MHR can be used as a good predictor of the occurrence of intracranial and extracranial atherosclerotic stenosis. In addition, age has been proven to be one of the most obvious independent factors of risk for the incidence of intracranial and extracranial atherosclerosis (17, 18), which is consistent with the results of this study.

The MHR is linked with cerebral atherosclerotic stenosis. However, there are few studies on the correlation between the MHR and the occurrence or degree of extracranial and intracranial atherosclerotic stenosis. From the analysis of the mild, moderate, and severe stenosis groups, this study concluded that the MHR significantly affects the degree of stenosis. Chen (16) found that monocytes are closely relevant to the degree of peripheral atherosclerosis stenosis. A population-level study of arterial atherosclerotic ischemic stroke in southern China found severe HDL with carotid artery stenosis in the brain [cervicocerebral atherosclerotic stenosis (CCAS)] (19). An elevated level reflects increased degrees of inflammation and oxidative stress and an increased severity of coronary artery stenosis (4, 20).

Domestic and international studies have found ethnic differences in the frequencies of extracranial and intracranial atherosclerotic stenosis. In Europe and the United States, extracranial artery stenosis is the dominant stenosis, while in Asia, intracranial arterial stenosis is more common (21, 22). However, in this study, the rate of extracranial artery stenosis was slightly greater than that of intracranial stenosis, which is consistent with the increasing prevalence of extracranial artery stenosis in Chinese people revealed by epidemiological surveys in the recent years (23). The higher rate of extracranial artery stenosis than of intracranial stenosis in this study may be due to the following factors: (1) Regional, dietary, and lifestyle differences. In high-altitude areas, the temperature difference between day and night due to the cold climate can limit the availability of fruits and vegetables. In addition, the dietary habits of the population include a high intake of meat, which can increase blood lipid levels and ATP (as measured by the plasma arteriosclerosis index). Moreover, long-term exposure to a hypoxic environment changes the blood microcirculation, anatomy, and physiology (24). (2) Aging with the proportion of stenosis cases involving intracranial arteries decreases, while the proportion of those involving extracranial arteries increases (25). China's Aging Society may exacerbate this phenomenon.

Conclusions vary with respect to the factors that influence the intracranial vs. extracranial distribution of atherosclerotic stenosis. This study concluded that male sex and smoking are independent risk factors for extracranial atherosclerosis alone, which is consistent with previous large-sample data studies (22, 26). Men are more prone to intracranial and extracranial atherosclerosis than women, which reflect the protective effects of estrogen on the cardiovascular and cerebrovascular systems such as its direct effect on the vascular wall and its beneficial effects on lipid composition. Estrogen resptor alpha 36 (ERa36) and estrogen receptor G protein-coupled receptor 30 (ERGPR30)/G protein coupled estrogen receptor (GPER1) signaling has been found to play an anti-inflammatory role in monocyte-/macrophage-related inflammatory processes (27). Recent studies have shown that estrogen can activate the GPER signaling pathway, which results in decreased SR-BI expression in endothelial cells and, thus, significantly reduces the transport of LDL-C (28). Furthermore, estrogen can inhibit liver esterase activity, improve the level of circulating HDL-C, reduce blood cholesterol and LDL-C, and directly interact with HDL-C to inhibit the oxidation of LDL-C, thus preventing atherosclerosis (29). However, diabetes mellitus was not found to be associated with extracranial or intracranial atherosclerotic stenosis, which is in contrast to previous results indicating that diabetes is a factor of risk for intracranial artery stenosis (24, 26, 30). The results may be due to the following: (1) Chronic hypoxic acclimatization at high altitude increases the dependence of the body on glucose and enhances glucose utilization, (2) With the improvement in standards of living of the residents, the incidence of diabetes mellitus has been rising rapidly. Diabetes mellitus has been shown to increase the incidence and burden of vascular risk factors and is common in both the intracranial and extracranial atherosclerotic stenosis (31, 32). This study concluded that age is an independent risk factor for intracranial and extracranial atherosclerosis and previous work has shown that the incidence of cerebral artery stenosis rises significantly with age (25). It is generally believed that the occurrence of extracranial artery stenosis is more strongly correlated with age than that of intracranial arterial stenosis (22, 30). However, a postmortem report (33) showed that the frequency of intracranial arterial stenosis increased with age. In addition, the results of this study suggest that lymphocytes might have protective effects against intracranial arterial stenosis. Recent studies have found that the number of circulating lymphocytes is significantly reduced in the progression of atherosclerotic lesions, which may be related to weakened adaptive immunity and healing effects in the atherosclerotic process (3, 34). The number of lymphocytes is highly related to the presence of extracranial artery stenosis. The lack of elastic fibers in intracranial vessels, the dense internal elastic layer, and increase in antioxidant enzyme activity with age provide good barrier effects. Intracranial atherosclerotic stenosis appears later than extracranial atherosclerotic stenosis and lymphocyte values are reduced in intracranial arterial stenosis (17). This study found that an elevated MHR value was related to significantly raised risks of simple intracranial arterial stenosis, combined extracranial and intracranial arterial stenosis, and simple extracranial arterial stenosis. The identification of the MHR as a common independent correlated factor in the ICAS, ECAS, and I-ECAS groups confirmed the MHR to be closely related to cerebral atherosclerotic stenosis.

Although the “gold standard” of cerebrovascular DSA examination was used in this study to diagnose intracranial and extracranial atherosclerotic stenosis, this method is traumatic, risky, and costly; thus, its use is mainly limited to the subset of patients with cerebral infarction who require surgery. For the patients in this study, DSA was found to be reliable for determining the stenosis rate and to have good precision and other advantages. However, patients with mild or no symptoms who did not opt for cerebrovascular DSA examination could not be included in this study. Thus, the total sample scale should be increased in future studies to verify the present results. Moreover, this study was limited to patients in plateau regions. In addition, data on the long-term (6 months or longer) clinical outcomes of patients are crucial to enhance the use of the MHR. Thus, follow-up clinical control studies should be conducted at multiple centers and regions to confirm the findings.

## Conclusion

In conclusion, as a risk factor for extracranial and intracranial atherosclerotic stenosis, the MHR has predictive value and is highly related to the severity and location of stenosis. This study also expounds on the development of extracranial and intracranial atherosclerotic stenosis in the process of inflammation, providing a theoretical basis for targeted interventions. Such interventions would reduce the incidence of cerebral atherosclerotic stenosis caused by ischemic cerebrovascular disease and provide better health services to the residents of high-altitude areas.

## Data Availability Statement

The original contributions presented in the study are included in the article/supplementary material, further inquiries can be directed to the corresponding author/s.

## Ethics Statement

The studies involving human participants were reviewed and approved by Ethics Committee of Qinghai Provincial People's Hospital. The patients/participants provided their written informed consent to participate in this study.

## Author Contributions

ZL, SW, and QF performed the study design, interpretation of the results, and statistical analyses. YL participated by analyzing and resolving difficulties of analytic strategies and the discussion. QF performed the final review and is the corresponding authors. All authors have approved the final manuscript after reading.

## Funding

This study was supported by grants from the Clinical Medical Research Center Project of Qinghai Province (Grant #2017-SF-L1) and the Qinghai Provincial Health Commission Project (Grant #2020-wjzdx-29) program funds. We confirm that any aspect of the work in this manuscript involving human patients was carried out with the ethical approval of all the relevant agencies.

## Conflict of Interest

The authors declare that the research was conducted in the absence of any commercial or financial relationships that could be construed as a potential conflict of interest.

## Publisher's Note

All claims expressed in this article are solely those of the authors and do not necessarily represent those of their affiliated organizations, or those of the publisher, the editors and the reviewers. Any product that may be evaluated in this article, or claim that may be made by its manufacturer, is not guaranteed or endorsed by the publisher.
